# Severe Hyperostosis Frontalis Interna in a 91-Year-Old Female Cadaver: A Case Report

**DOI:** 10.7759/cureus.79234

**Published:** 2025-02-18

**Authors:** Josephine Chiu, Jason Nikirk, Mario Loomis, Sean Hu, George Prada

**Affiliations:** 1 Clinical Anatomy, Sam Houston State University College of Osteopathic Medicine, Conroe, USA

**Keywords:** bone growth, case report, class d hfi, cranial pathology, hfi, hyperostosis, hyperostosis frontalis interna, postmenopausal woman

## Abstract

Hyperostosis frontalis interna (HFI) is characterized by benign thickening of the frontal bone and most commonly affects postmenopausal elderly women. While the exact cause remains unknown, its prevalence is thought to be multifactorial. This case report presents a discovery of extensive HFI during routine dissection of a 91-year-old female cadaver in the Clinical Anatomy Laboratory of Sam Houston State University College of Osteopathic Medicine. The patient's (donor) death was reported as systolic heart failure, and complete bilateral invasion of the frontal and temporal bones, with lesser involvement of the parietal bone, was observed. This condition extended from the crista galli anteriorly to the coronal suture posteriorly and to the sphenoid bone (SB) inferiorly. The greater and lesser wings of the SB were covered by the hyperostotic processes and the foramina (rotundum, ovale, spinosum, and lacerum) bilaterally. The cranial vault demonstrated an irregular surface with numerous nodular, trabeculated, and spiculated bony projections, creating a cobblestone-like appearance, a feature commonly referred to in pathology as bosselated lesions. A mild expansion into the superior sagittal sinus (SSS) and the dura mater was observed, which is uncommon for this condition. Based on postmortem pathology, a suspected diagnosis of advanced type D HFI was made. This report discusses a unique case of HFI that may contribute to a better understanding, classification, evaluation, and treatment of patients affected by this condition.

## Introduction

Hyperostosis frontalis interna (HFI) is a benign thickening of the inner table of the frontal bone. It was first described by Morgagni in 1769 as a specific disorder characterized by "bone accretion" on the inner surface of the frontal bone with sparing of the midline [1,2]. She et al. suggest that as much as 5-12% of the population have HFI [3]. Based on the bony overgrowth of over 50% of the frontal bone, the HFI presented in this report is a suspected type D category HFI according to the morphological findings suggested by Hershkovitz et al. [4].

The condition commonly affects postmenopausal elderly women with earlier and more rapid incidences in African Americans [4,5]. Clinical manifestations, including headache, orientation deficits, impaired memory, behavioral changes, and depression, have been reported in more than 20% of patients [5]. While the exact cause of HFI is unknown, it is believed to be associated with increased estrogen and leptin levels leading to an increase in bone mineral density over time [4]. It is also frequently related to hyperprolactinemia and elevated growth hormone [6]. This may explain the excess bony growth and angiogenesis or the link to obesity in patients with HFI [7-9]. Another theory is that HFI is due to a "profound change in human fertility patterns, introduction of hormonal therapy, and diet" [10]. Typically, HFI is asymptomatic, but surgery may be indicated if complications, such as intracranial hypertension and ischemia, arise [11]. 

This paper reports a severe case of type D HFI identified in the postmortem pathology of a 91-year-old female cadaver, featuring abnormal bone growth extending from the frontal bone into the temporal and sphenoid bones (SB), with minimal invasion of the parietal bone and involvement of the superior sagittal sinus (SSS). This case is unique because reported HFI is usually confined to the frontal and temporal bones, typically without crossing into the SSS.

## Case presentation

**Figure 1:**
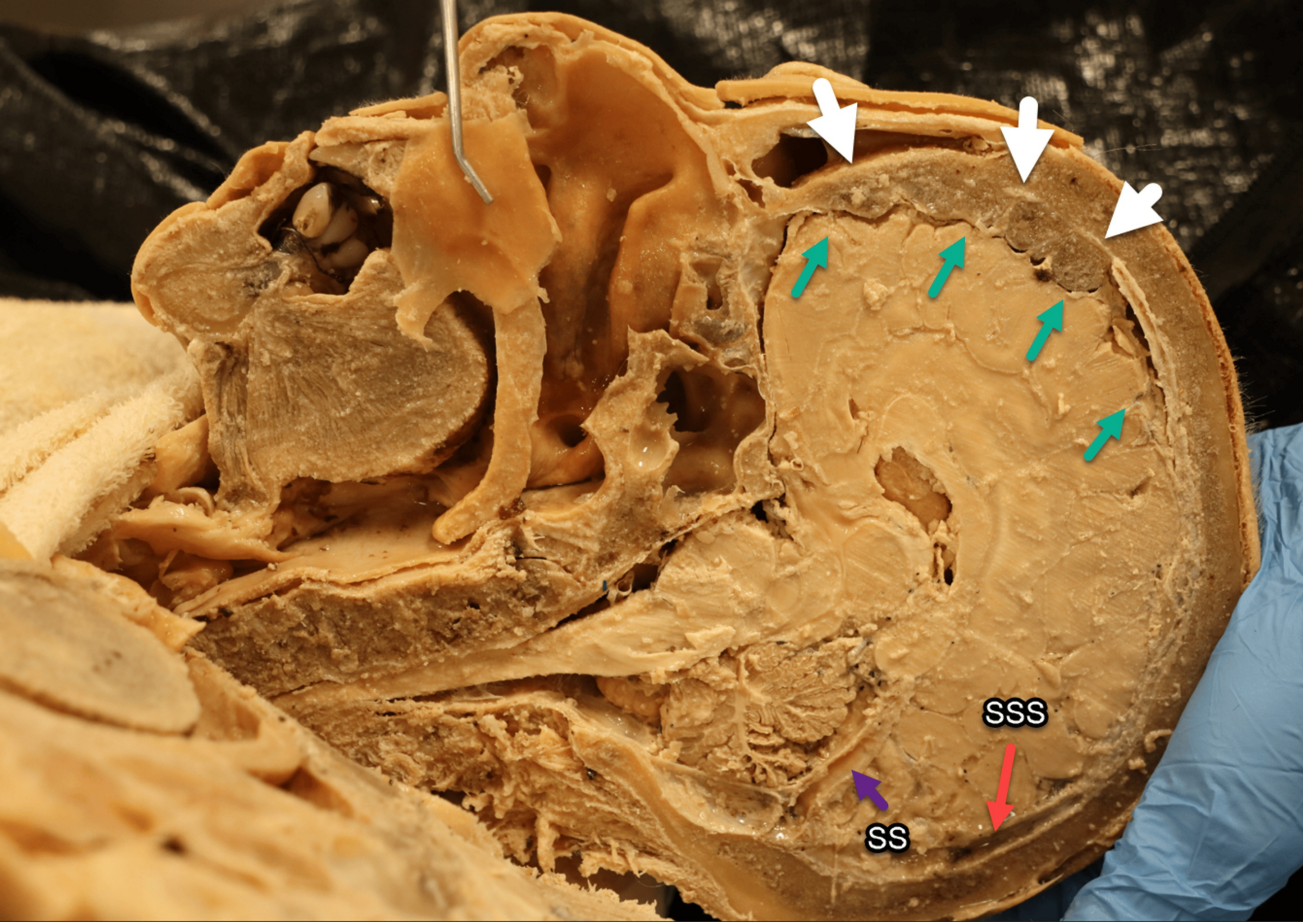
Hemisected head (right side). Hyperostotic nodules (white arrows) causing frontal lobe compression (green arrows) with the involvement of the SSS and dura mater (white arrows). SS shows no invasion (purple arrow). SSS, superior sagittal sinus; SS, straight sinus

A case of HFI was discovered during a routine dissection of a 91-year-old Caucasian female cadaver. The reported cause of death was systolic heart failure. Hemisection of the head was performed using an electric bone saw. A bony mass compression on the right frontal lobe of the brain was visible in its entirety (Figure 1). The pathology involved and obliterated approximately 25% of the SSS with osseous thickening formation on this region.

**Figure 2:**
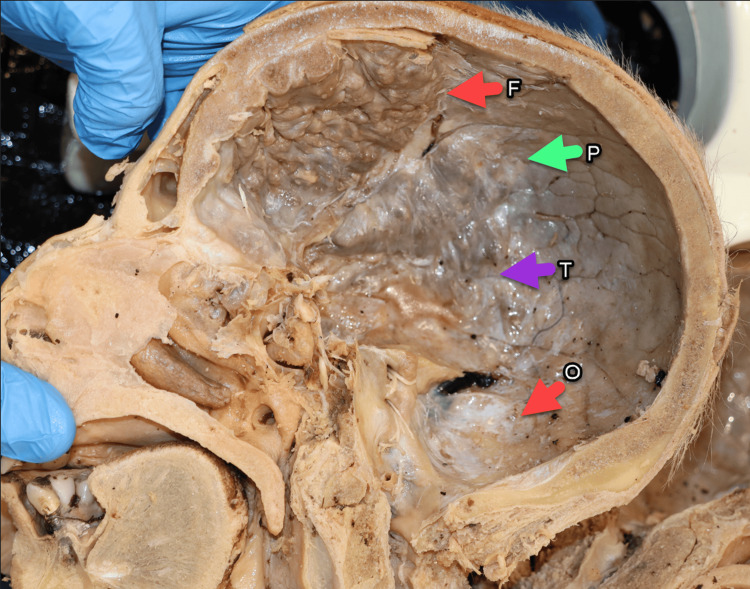
Severe HFI case. Hemisected skull (right side) showing frontal (F), parietal (P), temporal (T), and occipital (O) bones. HFI, hyperostosis frontalis interna

The frontal and temporal bones were mostly affected while the parietal bone showed a preliminary ongoing process (Figure 2). Coalescing nodular bony overgrowths, measuring between 0.5 and 1 cm in thickness, were observed along the inner table of the frontal bone. These growths primarily extended into the sphenoid and temporal bones, while the parietal bone remained mostly unaffected. 

**Figure 3:**
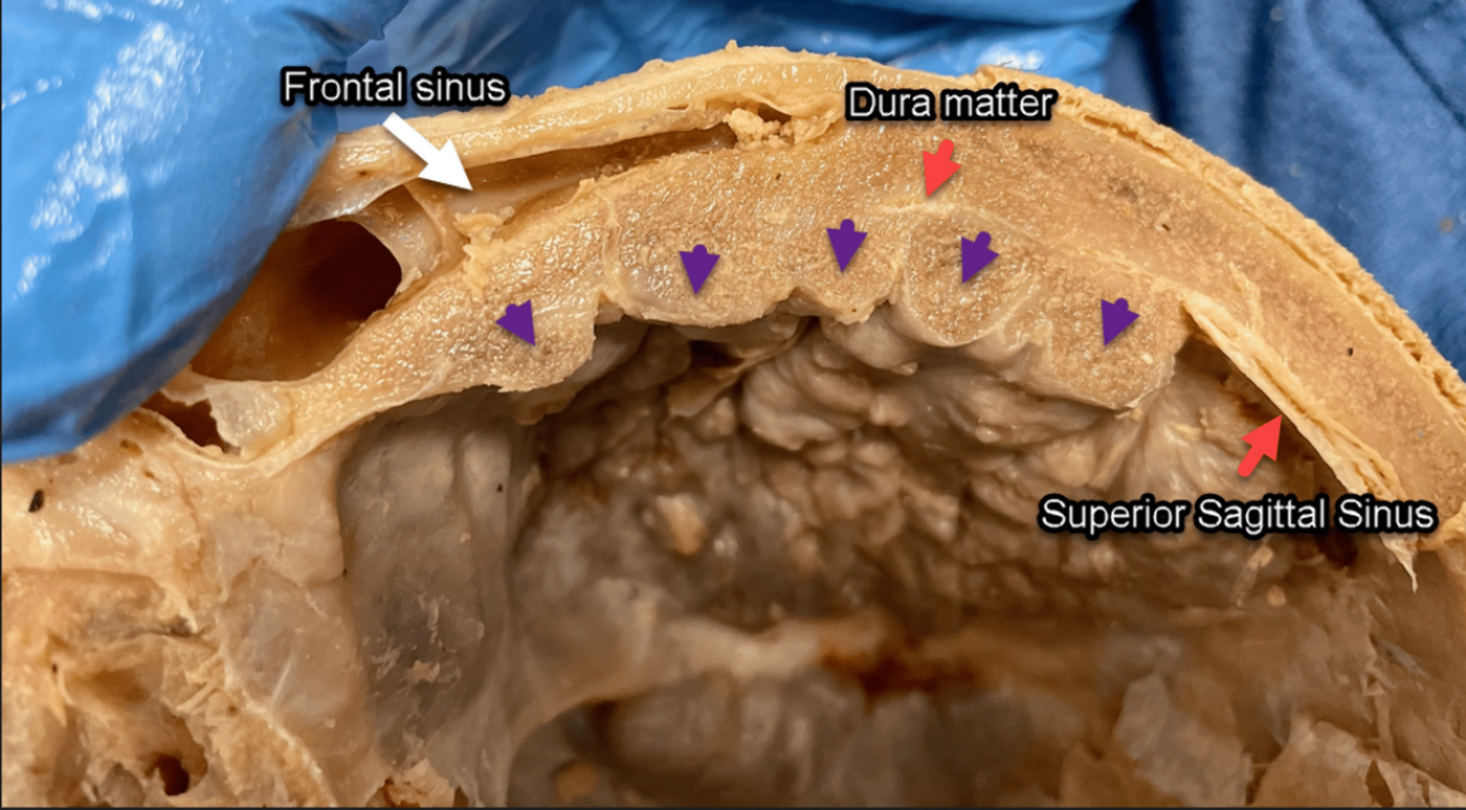
Nodular formation (purple arrowheads). Dura mater and SSS (red arrows) are overgrown and obliterated by the nodular formation. SSS, superior sagittal sinus

The dura mater was embedded between the frontal inner table and the hyperostotic nodular formations, which occupied the roof, floor, and lateral inner surface of the frontal bone (Figure 3). The bony accretion was characterized as irregular, rough, spiculated, and nodular. This growth compressed the entire frontal and temporal lobes with minimal compression of the parietal lobe. Compression of the SSS, arachnoid granulations, and bridging veins was also observed. This mass exerting pressure over various portions of the brain may have resulted in a range of clinical symptoms in the patient, though no clinical information was available to corroborate this.

**Figure 4:**
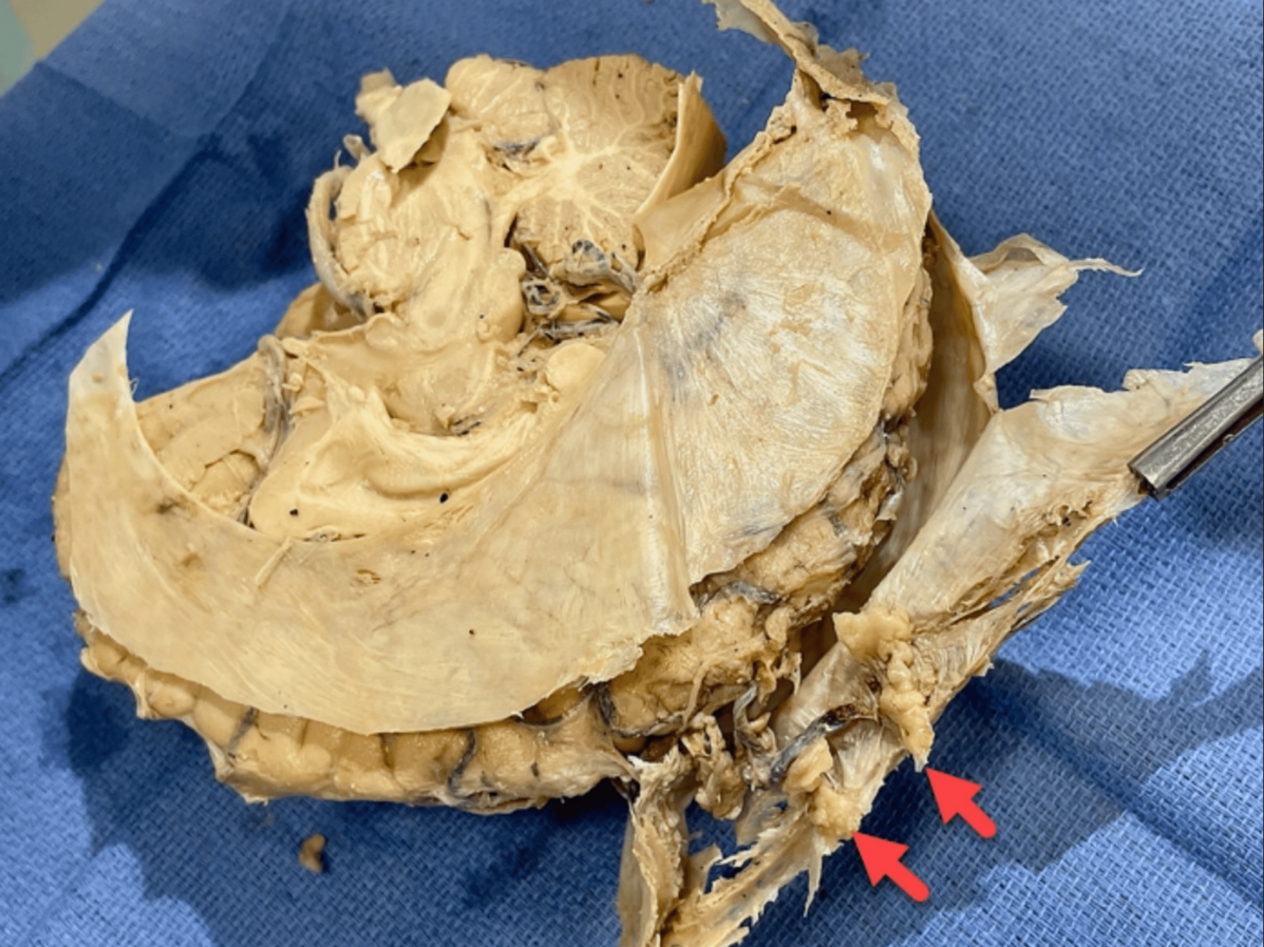
Left hemisphere of the brain with ossification of the falx cerebri dura mater (red arrows).

Both hemispheres of the brain were removed for gross examination. Calcifications on the superior border of the falx cerebri were noted on the left hemisphere of the brain, which may possibly be due to age (Figure 4). These calcifications were adherent to the dura mater. 

**Figure 5:**
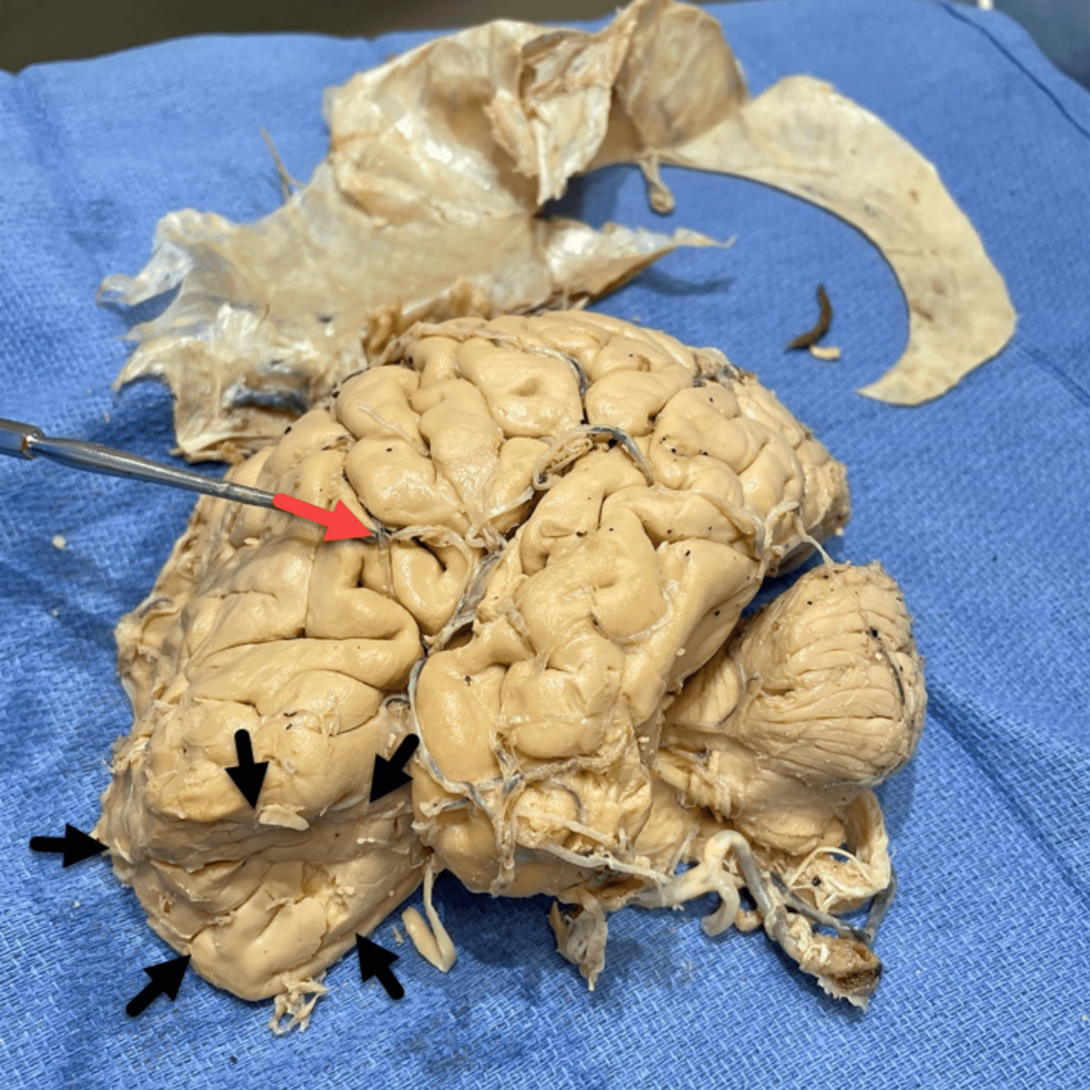
Left hemisphere of the brain. Deep frontal lobe compression (black arrows) and Broca's region (red arrow).

A deep mass compression was observed on the anteroinferior left frontal lobe and Broca's area (Figure 5). HFI is generally asymptomatic, but the significantly depressed area of the frontal lobe might very well have been associated with some speech irregularities, though we have no clinical confirmation of this.

**Figure 6:**
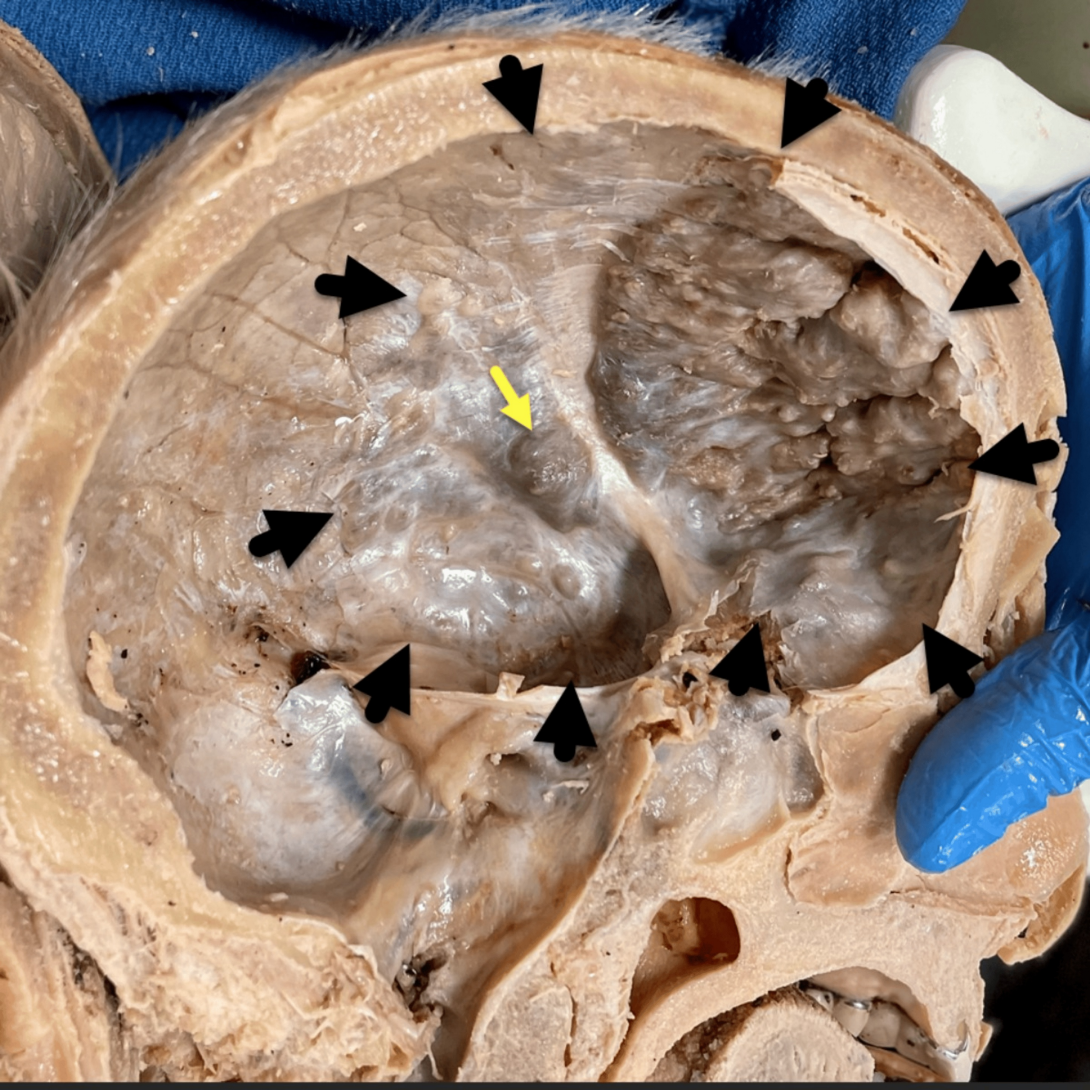
Hemisected skull (left side). Hyperostostic expansion (black arrows) within frontal and temporal bones and 1.5 cm nodular formation (yellow arrow) superior to Broca's area.

The inner table of the left frontal bone showed extensive, deep, and large nodular bone formations on the roof and floor, corresponding to the left frontal lobe depression, which extended to the medial side of the squamous part of the left temporal bone (Figure 6). A concentric hypertrophic protuberance, approximately 1.5 cm in diameter, was found medial to the pterion on the inner surface of the skull, at the junction of the inferior border of the parietal bone and the greater wing of the sphenoid bone (SB). This formation was compressing Broca’s area. 

**Figure 7:**
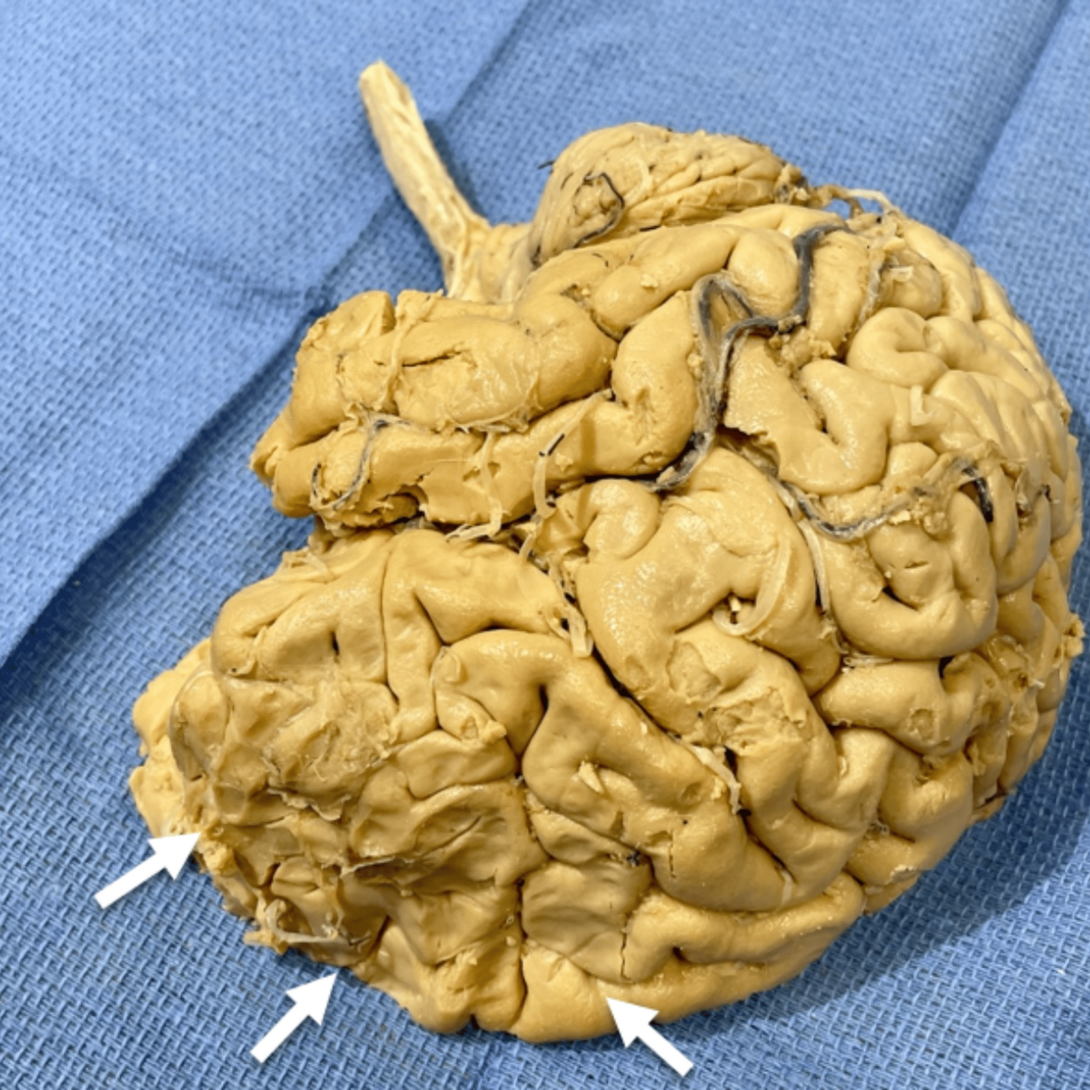
Right hemisphere of the brain with frontal lobe compression (white arrows).

The right frontal lobe demonstrated a continuous anterosuperior depression extending toward the central sulcus (Figure 7). These anomalies of the frontal lobe could have cognitive and behavioral implications, though this remains speculative.

**Figure 8:**
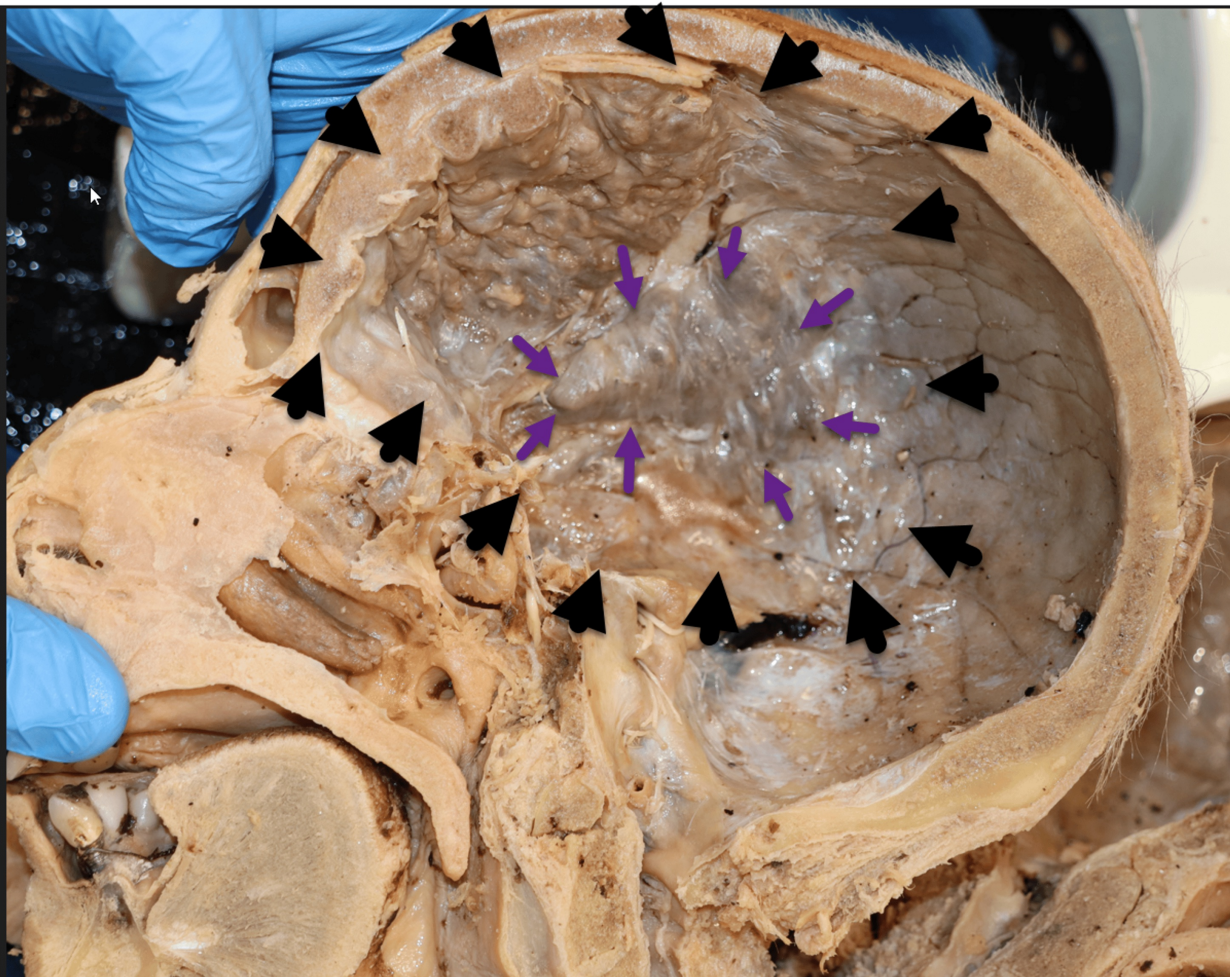
Hemisected skull (right side). Hyperostotic expansion (black arrows) within frontal and temporal bones with 5x4 cm triangular-shaped nodular formation (purple arrows). Black arrows show the perimeter of the hyperostosis process.

The inner squamous surface of the right temporal bone contained an approximately 5x4 cm triangular-shaped nodule compromising the right homolog of Broca’s area (Figure 8).

**Figure 9:**
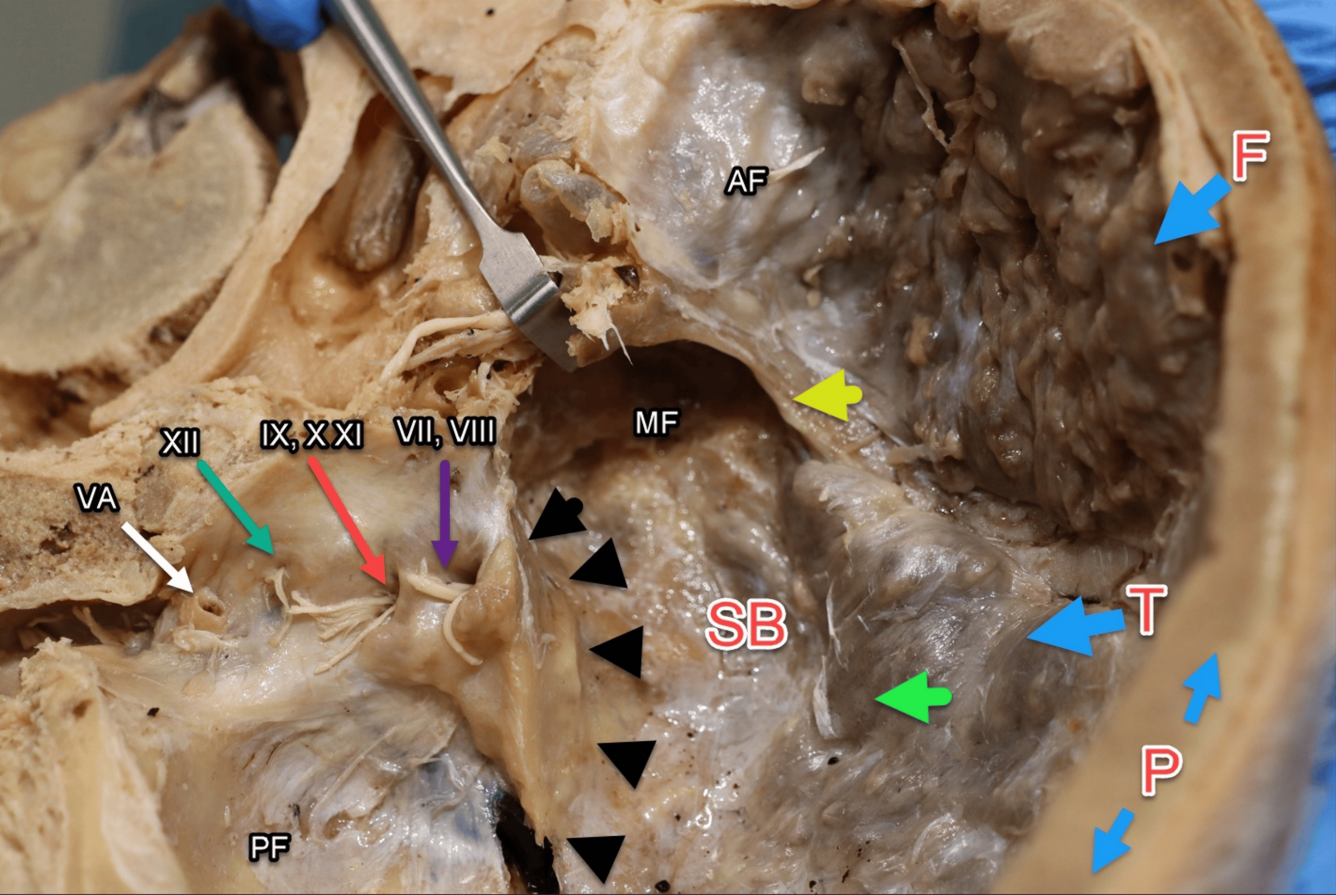
Cranial fossa overview. Illustration of the AF, MF, and PF, with the PF largely spared. Key bones include the frontal bone (F), parietal bone (P), and SB. The lesser wing (yellow arrow) and greater wing (green arrow) of the sphenoid bone are highlighted. Cranial nerves shown include facial (CNVII), vestibulocochlear (CNVIII), glossopharyngeal (CNIX), vagus (CNX), accessory (CNXI), and hypoglossal (CNXII). Black arrows indicate the petrous bone. SB, sphenoid bone; AF, anterior cranial fossa; MF, middle cranial fossa; PF, posterior cranial fossa

Within the anterior cranial fossa, the ethmoid bone and crista galli were buried by the hyperossification, including the cribriform foramina through which the olfactory nerve (CNI) enters (Figure 9). The hyperostosis lesions advanced inferiorly into the middle cranial fossa with complete ossification over the greater and lesser wings of the SB, covering the foramen rotundum, ovale, spinosum, and lacerum as well as the superior orbital fissure and optic canal. This pathology may have potentially compromised the maxillary (V2) and mandibular (V3) branches of the trigeminal nerve (CNV), internal carotid artery, deep petrosal nerve, and greater petrosal nerve [12]. The ophthalmic (CNII), oculomotor (CNIII), trochlear (CNIV), and abducens (CNVI) nerves may also have been involved. The potential clinical implications of nerve involvement and ossification of these foramina during the patient's life cannot be confirmed. This nodule also covers the squamous inner surface of the temporal bone with minimal invasion of the parietal bone. The petrous portions of the temporal bone were bilaterally spared by this condition, including the internal acoustic meatus, jugular, and hypoglossal foramina with their respective cranial nerves (VII, VII, IX, X, XI, and XII) and the vertebral artery. 

**Figure 10:**
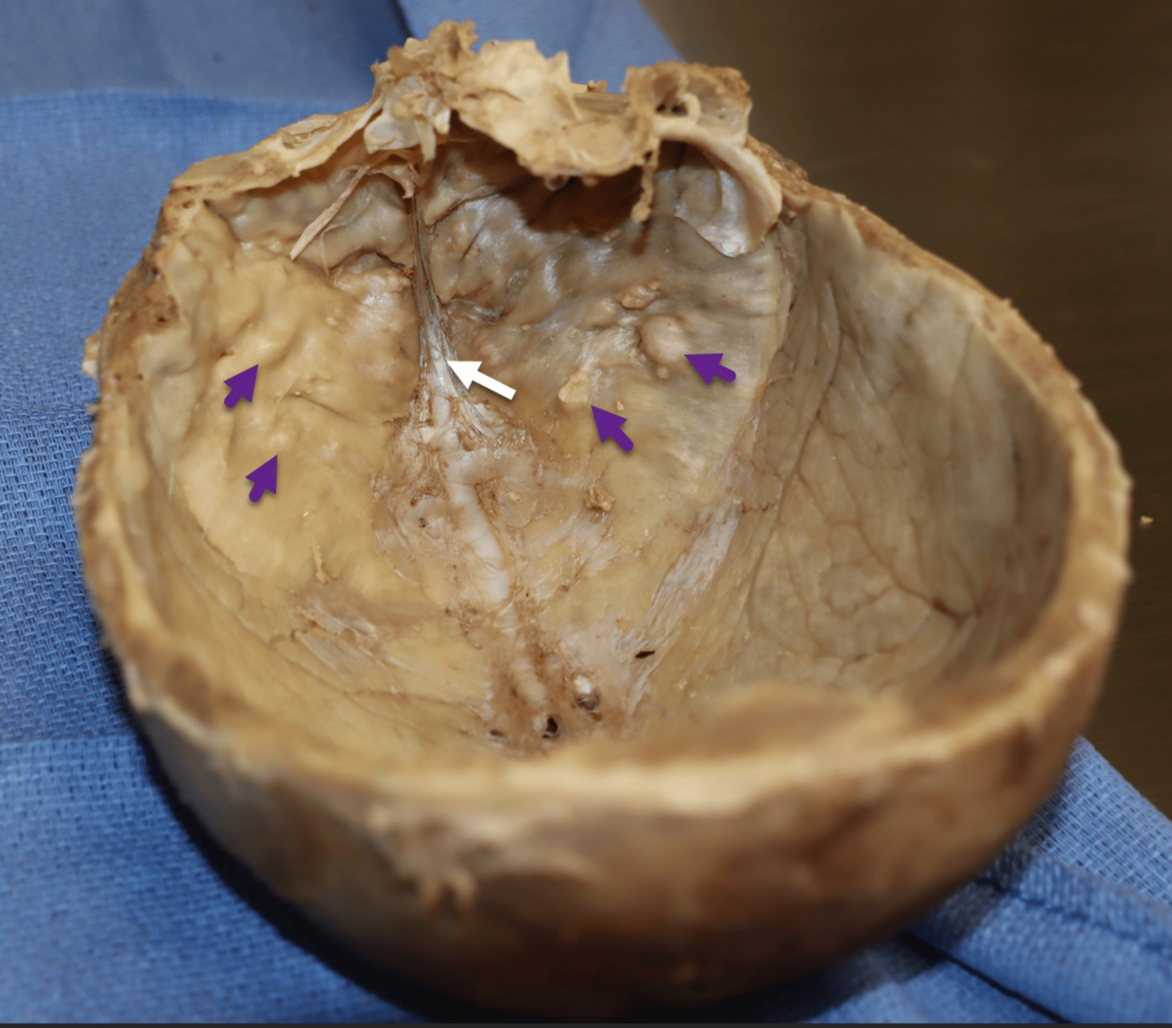
Skull of a 90-year-old Caucasian female with mild HFI, showing sparing of the midline (white arrow) and nodular formation on the inner surface of the frontal bone bilaterally (purple arrows). HFI, hyperostosis frontalis interna

The calvaria of another 90-year-old female cadaver who died from congestive heart failure was obtained for comparison, which displayed mild HFI invasion with sparing of the midline (Figure 10). Isolated bony growths and nodule formation were observed bilaterally on the surface of the frontal bone. The dissection procedures were conducted at the same institution.

## Discussion

Hershkovitz classified HFI according to the magnitude and manifestation as type A through D. Type A is generally under 10 mm in size, with elevated bony islands commonly found on the anteromedial part of the frontal bone. Type B consists of nodular bony overgrowths with only slight elevation, identified on less than 25% of the frontal bone. Type C involves more extensive nodular bony overgrowth, with up to 50% invasion of the frontal bone, and Type D involves more than 50% of the frontal bone, irregularly elevated with sharp, clearly demarcated borders [4]. This report presents a highly unusual case of Hershkovitz type D HFI: continuous nodular bony formations involving more than 50% of the frontal endocranium surface. The extensive bilateral thickening of the frontal bone with invasion into the temporal and parietal bone along with possible SSS growth is highly suggestive. The differential diagnosis for other skull pathologies was considered. Meningiomatosis and chordoma could not be ruled out, and histological analysis was not possible during this study. However, the likelihood of meningioma is low, as the tumor would typically exhibit inward growth rather than peripheral involvement of the bone. Paget’s disease of bone may involve the skull; however, widening of the diploe space would be expected, which was not seen in this specimen. Acromegaly was additionally ruled out due to the lack of enlarged features suggestive of this pathology. HFI is also associated with a variety of syndromes such as sclerotic bone metastases, Morgagni-Stewart-Morel Syndrome (HFI, obesity, and excessive hair growth/hirsutism), Froelich Syndrome (HFI, obesity, growth retardation, and pituitary dysfunction), and Troell-Junet Syndrome (HFI, acromegaly, diabetes mellitus, and toxic goiter) [13]. Despite these associations, Hershkovitz suggests that there is insufficient evidence to confirm whether HFI is strictly related to a specific syndrome, as the condition is commonly an incidental finding in elderly females [4]. Our specimen was not obese and had no indications of excessive hair growth. She et al. also note that their specimens were obese with comorbid diabetes mellitus but did not display hirsutism [3]. Morita et al. noted their specimen was not obese nor experienced hirsutism or any signs that could cause HFI [14]. 

The etiology of HFI is unclear. Ruhli and Cvetkovic indicate a potential link to endocrinological abnormalities as suggested by the greater prevalence of HFI in postmenopausal women [6,9]. While it is rare, HFI can occur in men as well. Yamakawa et al. presented a case of a 72-year-old man with hypogonadism characterized by underdevelopment of the penis and testes (despite preserved fertility), absent axillary hair, markedly reduced testosterone, and elevated luteinizing hormone [15]. This case further contributes to the speculation that HFI is correlated with endocrine abnormalities. A nutritional etiology has also been proposed, linked to modern dietary changes, easier access to foods, along with higher metabolic rates and leptin levels. Increased consumption of phytoestrogens such as soy and certain vegetables (broccoli, edamame, etc.) over the last few centuries may explain the commensurate rise in estrogenic levels. This explanation aligns with HFI predominance in females or feminine endocrinology. While the role of estrogen and its dysregulation is routinely seen in postmenopausal women with HFI, the complex relationship between environmental and biological factors in HFI still requires further investigation [16]. 

Despite uncertainties regarding its etiology and prognosis, hyperostosis frontalis is generally considered a benign condition and is most often an incidental finding during imaging of the skull [1]. Several studies acknowledge that the most common symptom in all cases was headaches [8,17,18]. A blinded PRISMA-guided search of HFI case reports found that high-frequency clinical manifestations of HFI include headaches, obesity, vertigo, dizziness, cognitive decline, and depression [1]. It is believed that HFI occurs in up to 12% of the postmenopausal population. However, since the HFI pathology is largely asymptomatic, many cases may remain undiagnosed.

## Conclusions

Our case presents significant hyperossification of the inner table of the frontal bone, extending into the middle cranial fossa and SSS. Gross inspection revealed numerous compressions of the brain underlying these areas. Based on the Hershkovitz HFI classification system, we believe our specimen falls into the Type D category, and given the physical presentation of the patient along with the gross anatomical features, we consider this a novel report of HFI.

Due to the inability to obtain histological confirmation and the lack of the patient's past medical history, no definitive conclusions can be drawn regarding whether this patient may have suffered from a specific neurological syndrome or neurological complications due to the widespread distribution of the pathology. The extensive nature of the undiagnosed HFI in this case underscores the possibility of HFI as an unsuspected cause of neurological symptoms in the elderly in whom extensive radiologic workups might not usually be undertaken.
